# Surgical treatment of rectal cancer: prospective cohort study about good oncologic results and low rates of abdominoperineal excision

**DOI:** 10.1590/0100-6991e-20233435-en

**Published:** 2023-07-19

**Authors:** THAIS ANDRESSA SILVA FAIER, FABIO LOPES QUEIROZ, ANTÔNIO LACERDA-FILHO, RODRIGO ALMEIDA PAIVA, PAULO ROCHA FRANÇA, MARCELO GIUSTI WERNECK CORTES, ALEXANDRE RIBAS DE CARVALHO, BARBARA MARIA TAVARES PEREIRA

**Affiliations:** 1 - Hospital Felicio Rocho - Belo Horizonte - MG - Brasil; 2 - Hospital Biocor - Belo Horizonte - MG - Brasil

**Keywords:** Colorectal Surgery, Adenocarcinoma, Neoadjuvant Therapy, Proctectomy, Pelvic Floor, Cirurgia Colorretal, Adenocarcinoma, Terapia Neoadjuvante

## Abstract

**Objectives:**

the purpose of this study was to evaluate the outcome of rectal cancer surgery, in a unit adopting the principles of total mesorectal excision (TME) with a high restorative procedure rate and with a low rate of abdominoperineal excision (APE).

**Methods:**

we enrolles patients with extraperitoneal rectal cancer undergoing TME or TME+APE. Patients with mid rectal tumors underwent TME, and patients with tumors of the lower rectum and no criteria for APE underwent TME and intersphincteric resection. Those in which the intersphincteric space was invaded and in those with a free distal margin less than 1cm or a tumor free radial margin were unattainable underwent APE or extralevator abdominoperineal excision (ELAPE). We assessed local recurrence rates, overall survival and involvement of the radial margin.

**Results:**

sixty (89.6%) patients underwent TME and seven (10.4%) TME + APE, of which five underwent ELAPE. The local recurrence, in pacientes undergoing TME+LAR, was 3.3% and in patients undergoing APE, 14.3%. The local recurrence rate (p=0.286) or the distant recurrence rate (p=1.000) was similar between groups. There was no involvement of radial margins. Survival after 120 months was similar (p=0.239).

**Conclusion:**

rectal malignancies, including those located in the low rectum, may be surgically treated with a low rate of APE without compromising oncological principles and with a low local recurrence rates.

## RESUMO

**Objetivos:**

o objetivo deste estudo foi avaliar os resultados da cirurgia do câncer de reto, em uma unidade que adota os princípios da excisão total do mesorreto (ETM) com baixa taxa de amputação abdominoperineal (AAP).

**Métodos:**

os pacientes com câncer retal extraperitoneal foram submetidos a ETM ou ETM com amputação abdominoperianeal. Pacientes com tumores de reto médio foram submetidos a EMT e pacientes com tumores de reto inferior e sem critérios para AAP foram submetidos a EMT e ressecção interesfincteriana. Aqueles em que o espaço interesfincteriano foi invadido e naqueles com margem distal livre menor que 1cm ou margem radial livre de tumor foram inatingíveis foram submetidos a AAP ou excisão abdominoperineal extraelevadora (ELAPE). Avaliamos as taxas de recorrência local, sobrevida global e envolvimento da margem radial.

**Resultados:**

sessenta (89,6%) pacientes realizaram ETM e sete (10,4%) ETM + AAP, dos quais cinco realizaram ELAPE. A recidiva local, em pacientes submetidos a ETM com ressecção anterior baixa, foi de 3,3% e em pacientes submetidos a AAP, 14,3%. A taxa de recorrência local (p=0,286) ou a taxa de recorrência à distância (p=1,000) foi semelhante entre os grupos. Não houve envolvimento das margens radiais. A sobrevida após 120 meses foi semelhante (p=0,239).

**Conclusão:**

as neoplasias malignas retais, incluindo aquelas localizadas no reto baixo, podem ser tratadas cirurgicamente com baixo índice de AAP, sem comprometer os princípios oncológicos e com baixo índice de recorrência local.

## INTRODUCTION

Optimal treatment of rectal cancer has evolved considerably in recent decades. Surgery, in particular, has been standardized due to a better understanding of the mechanisms underlying tumor dissemination, and total mesorectal excision (TME) has become the main standard of care^1^ ^,^ ^2^. The key principle of TME surgery is pelvic dissection under direct vision in the peri-mesorectal plane facilitated by traction and countertraction, resulting in less autonomic nerve injury, optimal preservation of sexual and urinary functions, reduced perioperative bleeding, lower rates of local recurrence^3^ ^-^ ^5^ and higher rates of sphincter preservation^6^ ^,^ ^7^.

Major resectional surgery for rectal cancer incorporates two types of procedures, namely sphincter preserving restorative anterior resection or low anterior resection (LAR) and sphincter resection techniques with abdominoperineal excision (APE) and a permanent colostomy. APE is required for cases with direct invasion of the external anal sphincter, or when a surgical margin over 1cm cannot be attained. 

APE surgery, an apparently more radical procedure, has historically been associated with higher rates of local recurrence^7^, possibly due to patient or disease specific factors and not necessarily due to the surgical procedure itself. Nevertheless, the rate of APE with a permanent ostomy remains high. In the United States, APE is still carried out for advanced low rectal cancer in about 50% of patients. Many surgeons and other cancer specialists see APE as a radical procedure used to reduce the local recurrence rate. However, several papers from major centers have suggested that ideally APE should be done in no more than 15% of all cases of rectal cancer and that many of the cancers currently treated by APE, based on older concepts, could be treated by LAR using the ideas of TME with intersphincteric resection, no permanent colostomy and respect for oncological principles^1^ ^,^ ^2^ ^,^ ^4^ ^,^ ^5^ ^,^ ^8^.

The purpose of this study was to evaluate the outcome of rectal cancer surgery, with a focus on the local recurrence rate, at a unit that adopts the principles of TME with a high restorative procedure rate and a low APE rate. 

## METHODS

We prospectively gathered data on patients with extraperitoneal rectal cancer undergoing TME or TME+APE between January 1999 and December 2010 in the Department of Colorectal Surgery at the Felicio Rocho Hospital, a tertiary hospital center in Belo Horizonte - Brazil. Survival data were measured until 2020. The same staff surgeon supervised all procedures. The study included all patients aged over 18 years who underwent surgical treatment for adenocarcinoma of the rectum located up to 10cm from the anal margin measured by proctoscopy and/or magnetic resonance imaging (MRI). Patients undergoing partial mesorectal excision, multivisceral pelvic organ resection or palliative surgery were excluded. We also excluded patients with incomplete data and those who were lost to follow-up. All patients with a preoperative MRI staging of T3 or above (tumor invading the muscularis propria, the subserosa or nonperitoneal perirectal tissues) or an MRI showing N1 or N2 (metastasis to regional lymph nodes) were given neoadjuvant therapy with 5-fluorouracil - 350mg/m^2^/day + Leucovorin 20mg/m^2^/day for five days during the first and fifth weeks of radiotherapy (which consisted of 5040 cGy in 28 sessions). Patients receiving neoadjuvant therapy were operated between 6 and 8 weeks after the last radiotherapy session. Laparoscopic or open surgery was indicated at the discretion of the surgeon, based on patient-related issues such as the biotype and comorbidities. All patients received standard anesthetic care. Antimicrobial prophylaxis for Gram-negative and anaerobic bacteria consisted of a single dose of ceftriaxone 2g and metronidazole 1.5g thirty minutes prior to the skin incision. Prophylactic thromboprophylaxis with enoxaparin was prescribed for all patients. TME, as described by Heald et al.^3^, was the procedure of choice in patients with tumors of the middle and lower rectum. High ligation of the inferior mesenteric artery was done routinely. A protective ileostomy to divert colon transit was done in all cases in which the sphincter was preserved; it was closed after two months, on average. Patients with ultra-low tumors of the rectum, where APE was not indicated according to criteria that will be described below, underwent TME with intersphincterian resection to attain tumor-free distal and radial margins^9^ ^,^ ^10^. 

APE was indicated when adequate tumor-free margins with sphincter preservation was not possible, or when there was invasion of the external anal sphincter, or invasion of the internal sphincter where intersphincter disection was not indicated or unachievable, or if the interphincteric space was invaded. As of 2006, we started to carry out extralevator abdominoperineal excision (ELAPE) for patients in need of APE^9^ ^,^ ^10^. 

Patients were discharged from hospital after pain was adequately controlled with oral drugs, when they accepted oral food intake and when they learned how to care for the ostomy. Antithrombotic prophylaxis was given until the 28^th^ post-operative day.

The recurrence rate and overall survival at one, two, five and ten years was assessed in patients undergoing curative surgery. Postoperative mortality was defined as death within 30 days of surgery. We assessed radial margin involvement in all cases.

The Ethics Research Committee of the Felicio Rocho Hospital approved this study (CAAE 33642420.1.0000.5125) 

Nominal and categorical data were compared using the chi-square test, Fisher’s test and Monte Carlo simulation, when necessary. The Mann-Whitney test was used to assess the duration of hospital stay according to the type of surgery. The survival curves were estimated using the Kaplan-Meier method and the comparison of the curves by the Log-Rank test. In all tests, a significance level of 5% was applied. Analysis was done with the SPSS software IBM Corp. 2011, Armonk, NY.

## RESULTS

Eighty patients with extraperitoneal rectal cancer, located up to 10cm from the anal margin underwent oncological protectomy. Eleven patients that required palliative surgery were excluded and two patients were excluded due to incomplete registry data. The final sample consisted of 67 patients. Sixty patients (89.6%) underwent TME and seven patients (10,4%) underwent APE (2 patients) or ELAPE (5 patients). Table 1 shows these and other demographic data. Tumors in patients undergoing APE/ELAPE were significantly lower than those undergoing LAR (p=0.043) (Table 2). The average duration of hospital stay was nine days in patients undergoing LAR and 14 days for APE/ELAPE cases (p=0.460). The postoperative mortality rate was 4.45% (3/67), of which 14.3% (1/7) were patients undergoing APE/ELAPE and 3.3% (2/60) were patients undergoing LAR (p=1.000) (Table 3). 

**Table 1:** Data on tumor staging and type of surgery in patients undergoing curative surgery for rectal cancer (n=67).

		Type of Surgery			
		LAR (n=60)	APE + ELAPE (n=7)	n	p
Sex	Male	38 (63.3)	2 (28.6)	40	0.109*
	Female	22 (36.7)	5 (71.4)	27	
Age	<40 years	3 (6.5)	0 (0.0)	3	0.797**
	41 - 60 years	19 (41,3)	4 (57.1)	23	
	>60 years	24 (52.2)	3 (42.9)	27	
T	0	9 (15.0)	2 (28.6)	11	0.682**
	1	12(20.0)	1 (14.3)	13	
	2	15 (25.0)	2 (28.6)	17	
	3	21 (35.0)	1 (14.3)	22	
	4	3 (5.0)	1 (14.3)	4	
N	NX	3 (5.0)	0 (0.0)	3	0.709**
	Negative	43 (71.7)	5 (71.4)	48	
	Positive	14 (23.3)	2 (28.6)	16	
Stage	0	8 (13.3)	2 (28.6)	10	0.775**
	1	23 (38.3)	2 (28.6)	25	
	2	15 (25.0)	1 (14.3)	14	
	3	14 (23.3)	2 (28.6)	4	

NI: not informed; *chi-square test; **chi-square test with Monte Carlo simulation.

**Table 2:** Distance of rectal tumor from the anal margin in rectal cancer patients undergoing curative surgery (n=67).

Distance	APE + ELAPE		LAR		p- valor	OR	CI
	n	%	n	%			
5 to 10cm	1	14.29%	34	59,64%		0.036	0.002 to 0.585
2 to 5cm	4	57.14%	21	36.84%	0,043^1^	0.190	0.020 to 1.776
<2cm	2	28.57%	2	3.51%		1.000	-

^1^Fisher’s exact test; OR: odds ratio; CI: confidence interval.

**Table 3:** Mortality rate in rectal cancer patients undergoing curative surgery (n=67).

	Surgery				
Death		APE + ELAPE	LAR	Total	p-value
No	n	6	56	62	1.000*
	%	85.7%	93.3%	92.5%	
Yes	n	1	4	5	
	%	14.3%	6.7%	7.5%	
Total	n	7	60	67	
	%	100.0%	100.0%	100.0%	

*Fisher’s test.

The overall local recurrence rate was 4.4%. Local recurrence was seen in one case of patients undergoing APE (1/7) (14.2%) in whom the conventional abdominoperineal excision technique was used before the extralevator technique was adopted (Tabela 4). No local or distant recurrence was seen in the five patients undergoing ELAPE. Two local recurrences (3.3%) were seen in patients undergoing LAR. Five patients (8.3%) developed metastases, of which three were lung metastases, one was liver involvement and one had lung and liver metastases (Table 5). Pathology found no radial margin involvement in any of the specimens.

**Table 4:** Local recurrence rate according to type of surgery in rectal cancer patients undergoing curative surgery (n=67).

		APE + ELAPE			LAR	p-value	OR	CI
		n	%	n	%			
Local recurrence	Yes	1	14.2%	2	3.3%	0.286^1^	1.40	0.6 - 3.3
	No	6	85.8%	58	96.7%			

^1^Fisher’s exact test; OR: odds ratio; CI: confidence interval.

**Table 5:** Distant recurrence rates according to type of surgery in rectal cancer patients undergoing curative surgery (n=67).

		APE + ELAPE			LAR	p-value	OR	CI
		n	%	n	%			
Distant recurrence	Yes	0	0.00%	5	8.3%	1.000^1^	-	-
	No	7	100.00%	55	91.7%			

^1^Fisher’s exact test; OR: odds ratio; CI: confidence interval.

Survival at 12, 24, 60 and 120 months was respectively 93%, 91%, 81% and 73% in patients undergoing LAR, and 85.7%, 71.4%, 71.4% and 57.1% in patients undergoing APE/ELAPE (Tables 6 and 7 and Chart 1).

**Table 6:** Probability of survival in rectal cancer patients undergoing APE/ELAPE (n=7).

Time (months)	Number at risk	Probability of survival	Standard error (CI - 95%)
12	7	0.857143	0.132260 (0.5979 - 1.0000)
24	6	0.714286	0.170747 (0.3796 - 1.0000)
60	5	0.714286	0.170747 (0.3796 - 1.0000)
120	5	0.571429	0.187044 (0,.048 - 0.9380)

[Fig f1]

**Figure 1 f1:**
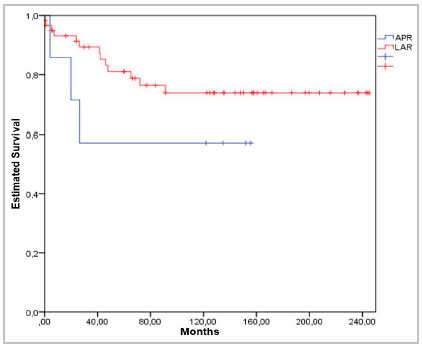
Estimated overall survival according to type of surgery in rectal cancer patients. (n=67).

## DISCUSSION

 In several colorectal surgery units throughout the world, the rate of abdominoperineal excision in patients with rectal cancer remains higher than what is recommended in the literature. Although the expected rate of APE and definitive colostomy in specialized units for the treatment of rectal cancer is around 15%, there are reports in the literature of AAP rates as high as 50% in these cases^2^. A retrospective study of 4,471 patients included in the database of the American College of Surgeon’s National Surgical Quality Improvement Program between 2016 and 2018 and conducted by Taylor et al.^8^ found APE rates in the US of impressive 52.2%. Lack of an adequate technique and the difficulty of performing a low colorectal or coloanal anatomosis meant that APE became adopted liberally until the end of the last century^2^.

After Heald^3^ standardized TME in 1982, developments in neoadjuvant therapy, surgical staplers and intersphincterian resection techniques resulted in significant improvements in the treatment of extraperitoneal rectal cancer, making it possible to resect low and ultra-low cancers without having to remove the sphincters and without compromising oncological safety^2^ ^,^ ^10^. In the present study, the overall rate of abdominoperinal excision and definitive colostomy was 10.4%; for cancers located up to 5cm from the anal margin, this rate was only 20.6%. Sphincter preservation was possible in four patients with cancers located up to 2cm from the anal margin. Even with such low rates of APE, our local recurrence rate (4.4%) and overall survival remained within norms established in the literature^12^ ^-^ ^14^. Surgical treatment with a low rate of abdominoperinal excision and with sphincter preservation was possible in rectal cancers located up to 10cm from the anal margin, without compromising oncological principles.

An important factor for a good oncological result is to attain a cancer-free radial surgical margin^15^. This becomes especially important in patients with lower cancers requiring AAP, as in the present study, where 85.7% of patients undergoing APE had cancers located less than 5cm from the anal margin. Only 38.3% of patients undergoing LAR had cancers in this site (p=0.043). The risk of radial margin involvement is higher in patients undergoing AAP because there is no mesorectal tissue at the sphincters, which leaves the radial margin more vulnerable^15^ ^,^ ^16^.

Heald et al.^4^ published a prospective study in 1986 that reported recurrence rates of 33% in patients undergoing conventional APE versus 1% in patients undergoing TME without APE. Marret al.^17^ also found significantly lower recurrence rates in patients undergoing LAR compared to those undergoing APE (23% vs. 13%). A recent multicenter study by Saito et al.^18^ consisting of 228 patients showed a local recurrence rate of 3.6% in patients undergoing LAR. 

A Brazilian study by Lacerda-Filho et al.^2^ evaluated 71 patients with rectal cancer who underwent LAR or APE, prior to the ELAPE era. Twenty patients had cancers in the lower third (28%) and 11 of them underwent APR (55%). Overall, 15.5 % of patients with rectal cancers were treated by APR. Estimated rates of local recurrence were 6% for LAR patients and 23% for APR patients (p=0.0778), as shown in that study. Those authors estimated that cancer-related survival rates were 67% in patients undergoing APR and 78% in patients undergoing LAR. They concluded that there was an important trend towards poor oncological results when using conventional APR compared with LAR in patients with rectal cancer. 

Holm et al.^19^, in 2007, described rectal excision using an extralevator approach (ELAPE) as an approach to overcome the limitations of APE surgery. This technique allows surgeons to obtain a robust cylindrical surgical specimen with a low probability of leaving residual disease in the pelvis. In the present study, no patient had compromised radial margins, and the overall recurrence rate was 4.4%. Seven APE procedures were carried out, two using the conventional technique and five using the extralevator approach. The only recurrence in this group occurred in a patient operated by conventional APE. Although histological proof of radial margin involvement was not found, a possible recurrence mechanism may have been a poor radial margin. There were no recurrences during follow-up in the five patients undergoing ELAPE. Notwithstanding the small number of patients in this study, this technique appears to yield better oncological results and should always be the preferred approach in patients with anteriorly located cancers that include the levator muscle and that have a higher risk of intraoperative perforation^19^.

The most important factor to be considered is that it was possible to perform an oncologically adequate surgery, with preservation of the sphincters in the vast majority of patients, even in those with low/ultralower lesions, avoiding a definitive colostomy. As shown in Table 4, the recurrence rate in the LAR group was 3.3%, with distant metastases being observed in 8.3% of patients (Table 5) and 5-year survival of 81% (Table 6), excellent results when compared to historical series in the literature^5^ ^,^ ^6^ ^,^ ^11^ ^,^ ^17^, showing that the adequate choice of the surgical technique and the performance of the surgery following the recommended technical standards, allows to preserve the anal sphincter, without harming the oncological results.

Preserving the sphincter in low and ultra-low rectal cancers is possible when associating TME and intersphincterian dissection to attain adequate distal and radial margins in ultra-low cancers, and using staplers or a manual coloanal anastomosis. Using the classical indications of APE would have resulted in at least 32.9% of our patients undergoing excision and remaining with a definitive colostomy. Of 29 patients with rectal cancer located up to 5cm from the anal margin (Table 3) sphincter preservation was possible in 79.4% (23/29) of these cases without compromising oncological results, by associating intersphincteric resection and TME. We also found similar overall 10-year survival rates in patients undergoing APE, compared to patients who had undergone LAR. 

Among the limitations of our study are a small sample, which does not allow an adequate statistical comparison between groups, and that fact that chemotherapy and radiotherapy at the time had poorer results compared to current therapies. Furthermore, the watch and wait approach for organ conservation was not as established then as it is currently, and was therefore not taken into account. Another aspect that was not considered, and that could enrich the study would be the assessments of function and the quality of life of these patients. 

We conclude that rectal malignancies, even low and ultra-low rectal cancers, may be treated surgically with low rates of APE without compromising oncological principles and with low recurrence rates. By standardizing the TME technique and associating it with transphincteric resection and neoadjuvant therapy, and by using adequate staplers, we were able to reduce the rates of APE.
